# Multi-Host Expression System for Recombinant Production of Challenging Proteins

**DOI:** 10.1371/journal.pone.0068674

**Published:** 2013-07-17

**Authors:** Steffen Meyer, Carmen Lorenz, Bahar Baser, Mona Wördehoff, Volker Jäger, Joop van den Heuvel

**Affiliations:** Department of Molecular Structural Biology, Helmholtz Centre for Infection Research, Braunschweig, Germany; New England BioLabs, United States of America

## Abstract

Recombinant production of complex eukaryotic proteins for structural analyses typically requires a profound screening process to identify suitable constructs for the expression of ample amounts of properly folded protein. Furthermore, the evaluation of an optimal expression host has a major impact on protein yield and quality as well as on actual cost of the production process. Here we present a novel fast expression system for multiple hosts based on a single donor vector termed pFlp-Bac-to-Mam. The range of applications of pFlp-Bac-to-Mam comprises highly efficient transient transfection of HEK293-6E in serum-free suspension culture and subsequent large-scale production of challenging proteins expressing in mg per Liter level using either the baculoviral expression vector system or stable CHO production cell lines generated by Flp-mediated cassette exchange. The success of the multi-host expression vector to identify the optimal expression strategy for efficient production of high quality protein is demonstrated in a comparative expression study of three model proteins representing different protein classes: intracellular expression using a fluorescent protein, secretion of a single-chain-Fv-hIgG1Fc fusion construct and production of a large amount of highly homogeneous protein sample of the extracellular domain of a Toll-like receptor. The evaluation of the production efficiency shows that the pFlp-Bac-to-Mam system allows a fast and individual optimization of the expression strategy for each protein class.

## Introduction

**Figure 1:**
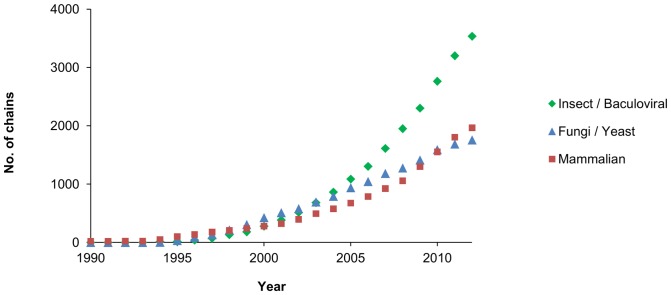
Total number of protein chains deposited in the PDB by expression host. Cumulative total number of protein chains in the PDB whose expression system was identified as mammalian, insect, baculovirus, fungi or yeast is plotted by year of deposition. Expression data were parsed from the set of PDB files available as of November 2012. Chains were counted rather than PDB entries as expression information is recorded by chains in the PDB.

The availability of large amounts of relevant target proteins in high quality is a prerequisite for structural analysis or drug-target screening strategies. Many viral and mammalian proteins either require specific post-translational modification for proper folding and full biological activity or have to be co-expressed in the presence of their partners to assemble in a multi protein complex for functional stability. As a result, *E. coli* is usually not suitable as an expression system for complex mammalian proteins. Therefore, eukaryotic expression hosts are indispensable for recombinant production of those proteins [1]. Thus, approximately 10% of all protein structures submitted to the Protein Data Base (PDB) have been solved after expression in eukaryotic systems. Therefore, facilities dedicated to mammalian protein expression like the Helmholtz Protein Sample Production Facility (PSPF) provide a broad spectrum of eukaryotic expression systems comprising yeasts, mammalian cell culture and the baculovirus expression vector system (BEVS). Due to their simple paucimannosidic *N*-glycosylation pattern and the scalability of the baculovirus-dependent expression in suspension culture lepidopteran cell lines are preferably used for the production of proteins for crystallization with a share of almost 50% among the eukaryotic systems (Figure 1). The prevalent mammalian host cell lines utilised for protein production are derived from the human embryonic kidney epithelia cell line HEK293 and CHO cells, which originate from ovaries of the Chinese Hamster. Among these, HEK293 is most commonly used for transient gene expression due to the availability of subclones adapted to suspension culture and an optimized genetic background for plasmid based transient expression [2]. Likewise, the development of effective transfection methods contributed to the success of this system. Using the cationic polymer polyethylenimine (PEI) as transfection reagent transfection rates of >80% can be achieved [3], [4]. Thereby, protein yields up to 1 g/L have been reported [5]. In contrast CHO cells lines are less suitable for transient protein expression, as only less than 30% of the yields achieved in HEK293 have been reported so far even though large efforts have been expended in the past years to optimise transient transfection of CHO cells [6]–[8]. However, they are predominately used for stable genomic expression as CHO cells integrate exogenous DNA with a high efficiency and genomic stability [9]. The glycosylation mutant cell line CHO Lec3.2.8.1 [10] is particularly suitable for the production of glycoproteins for structural analyses [11]. As previously reported the PSPF implemented an optimized system based on targeted integration via Flp-recombinase mediated cassette exchange (RMCE) [12] as an advanced and fast method to stably integrate target genes in this cell line to optimize our protein expression pipeline [13].

Despite all these improvements during the past decades protein production in eukaryotic cell lines from both invertebrate and vertebrate still is generally more time-consuming and expensive than in bacteria. Thus a profound screening for the best protein construct as well as the most appropriate host regarding both yield and quality of protein is essential. To address this, vectors for initial screenings harbouring promoters for different expression systems have been reported before [14], [15]. However, these plasmids suffer some major drawbacks that limit their usability in multiparallel expression studies in state-of-the art systems. For instance, they are not compatible to advanced transposition based techniques for the generation of recombinant bacmids [16] and novel systems emerged thereof such as MultiBac [17] or Acembl [18]. Moreover, they lack the EBVoriP for enhanced expression in optimised HEK293-6E cells and are not applicable for stable genomic expression in mammalian cells by the Flp-recombinase mediated cassette exchange system (RMCE).

In this report we present the construction and evaluation of the versatile shuttle vector pFlp-Bac-to-Mam (pFlpBtM) that can be used for both, fast transient and stable genomic expression in mammalian cells as well as a donor vector for the generation of recombinant bacmids. By the unique combination of genetic elements it streamlines the initial screening for expressible constructs and the most suitable host for any given protein. We demonstrate the applicability of this vector for the production of three different classes of eukaryotic model proteins. Accumulation of an intracellular model protein was validated by the expression of mCherry, a mutant of *Discosoma striata* red fluorescent protein [19]. A single-chain-Fv-hIgG1Fc fusion construct (scFv-Fc) [20] was used as a member of a well-known class of secretory therapeutic proteins that routinely are expressed with high-yields in mammalian cells. Additionally, the extracellular domain (ECD aa 1–578) of the murine Toll like receptor 2 was chosen as a second secreted model protein. As a member of the Leucine Rich Repeat (LRR) family of proteins this construct represents a challenging target protein for heterologous expression since it can only be produced in low amounts by using elaborate expression strategies [21].

## Materials and Methods

### Construction of pFlpBtM series of vectors

The polyhedrin promoter from pFastbac (Invitrogen) was exchanged by a PCR-Fragment of the hr5-ie1-p10 region from pIEx (Novagen) via BbsI-NsiI digest. After site directed mutagenesis to remove a BbsI-site within the promoter region, a PCR fragment containing the FRT-Cassette generated from the vector pFS-sighis-PGK (GenBank JF313343) flanked by BamHI at the 5′ end and AvrII at the 3′ end, was integrated into the modified pFastbac with the hr5-ie1-p10 promoter. The resulting intermediate construct (pFlpBtM-I, Genbank ID: KC991096) can be used as donor vector in BEVS and for RMCE. The final pFlpBtM-II vector (Genbank ID: KC991095) was constructed by replacing the hr5-ie1-p10 promoter region by a PCR-fragment harbouring the CMV-p10-T7 promoter region from pTriEx (Novagen). The backbone of the resulting vector was excised by SapI-EcoRV digestion and replaced by a PCR-fragment of a modified pTT5 backbone (NRCC) containing the EBNA1 oriP, a beta-lactamase gene and a pMB-ori. Prior to this integration both an NcoI and a BbsI site in the backbone of pTT5 were deleted by site-directed mutagenesis.

### Integration of Model Proteins

**Figure 2:**
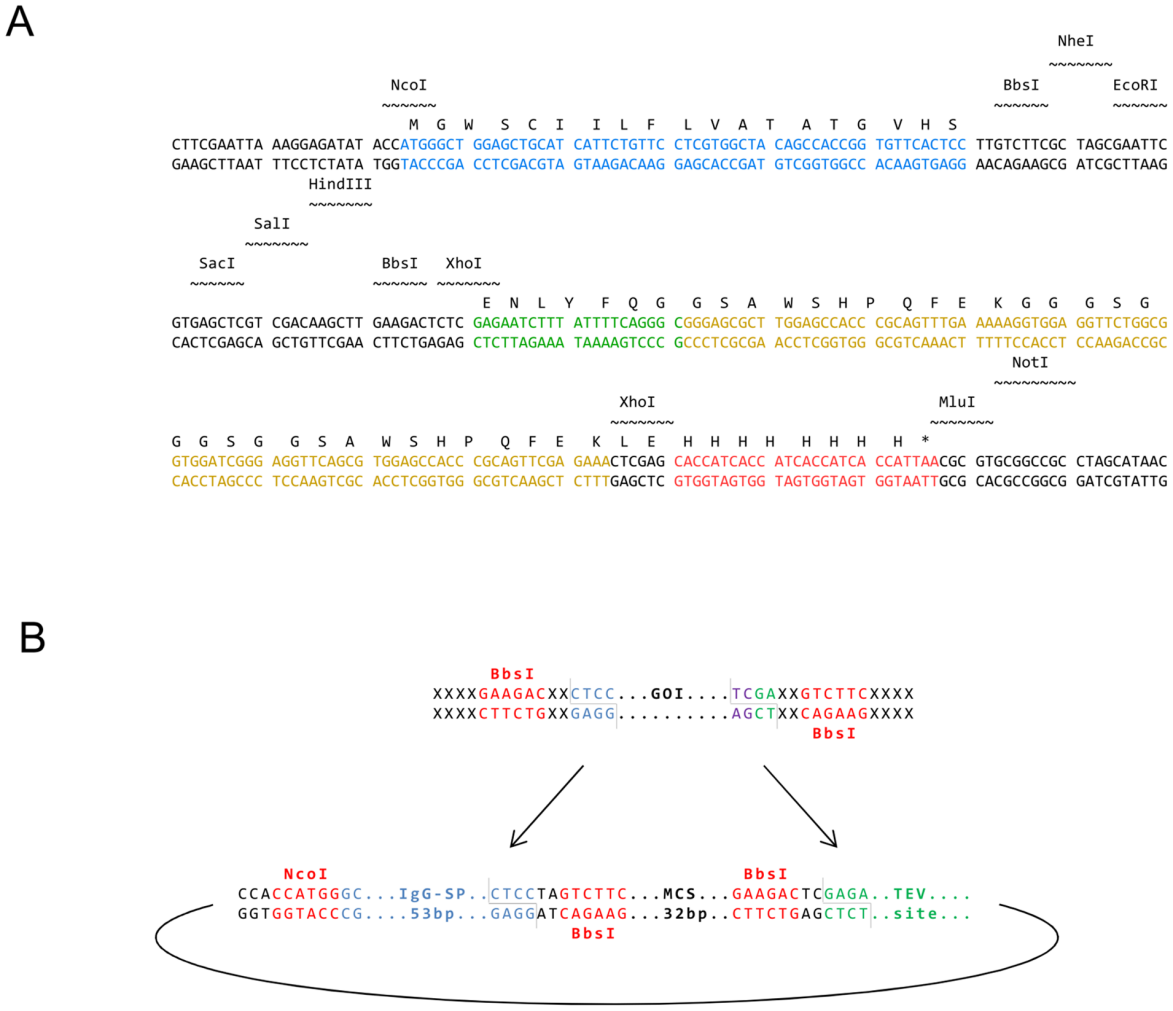
Multiple cloning site (MCS) and tags for purification and detection. Upstream of the MCS pFlpBtM harbours a human IgG signal peptide sequence (SP, blue) for the production of secretory proteins. Additionally, N-terminal Twin-Strep- (yellow) and 8xHis-Tags (red) are incorporated and separated by a TEV protease cleavage site (green) (**A**). In frame integration of genes can be performed using two BbsI sites oppositely between the SP and the TEV site. By the use of these non-palindromic Type IIS endonucleases a seamless in frame fusion to the 3′ affinity tags is generated. PCR fragment of the target gene flanked by BbsI or any other Type IIS restriction site of choice are necessary (**B**). The STrEP-One tag is additionally flanked by a pair of two XhoI restriction sites which allow for an integration of genes directely to the C-terminal 8xHis-tag by the excision of the Twin-Strep-tag. Likewise, NcoI can be used as the 5′ integration site to eliminate the IgG-signal peptide of the plasmid for intracellular accumulation or if secretion should be triggered by an authentic gene specific signal peptide. MluI or NotI can be employed as 3′-restriction sites to eliminate all N-terminal fusions.

For the intracellular accumulation of the model protein mCherry (gb AY678264), the corresponding gene was integrated into both pFlpBtM-I and pFlpBtM-II through a PCR-fragment flanked by 5′NcoI and 3′BbsI restriction sites. By cutting the pFlpBtM-II vector with NcoI the IgG-signal peptide (SP) is excised. Using the type IIS restriction enzymes BbsI for the integration of target proteins facilitates seamless in frame integration of the target protein to the C-terminal histidin tag of the vector (Figure 2). The secretory proteins ECD-mTLR2 (uniprot accession no. Q9QUN7, DNA sequence kindly provided by W.D. Schubert, University of the Western Cape, Africa) and a single-chain variable fragment fused to a human IgG1Fc (scFv-Fc, courtesy of T. Schirrmann, TU Braunschweig) were integrated with their authentic SP without generating fusions using 5′Nco and 3′XhoI or 3′Nhe respectively. All plasmids were purified by midi preps (Promega) and confirmed by sequence analyses.

### Transient Protein Expression in HEK293-6E

Cultivation of HEK293-6E [2] was performed in F17 medium supplemented with 25 mg/L G418, 1 g/L Pluronic F86 and 7.5 mM L-glutamine in an Infors Minitron orbital shaker at 100 r.p.m. at 37°C in a 5% CO_2_ atmosphere. For transfection 0.3×10^6^ cells/mL were seeded in a total volume of 22.5 mL in shake flasks (Corning) 3 days prior transfection. 2 µg DNA including 5% pTToeGFP as transfection control per 1e6 cells were diluted in 1.25 mL Medium and incubated with linear PEI (Polysciences #23966) in a PEI:DNA ratio of 2∶1 for 20 min at room temperature, before adding the PEI:DNA polyplexes to the cells. 24 h post transfection the cells were expanded to a total volume 50 mL volume and supplemented with 0.5% Tryptone N1 (Organotechnie #19553). 72 h post transfection the cells were fed with 4.5 g/L glucose. The expression of the target protein was enhanced by adding 3.75 mM valproic acid (96 h, post transfection).

### Stable Protein Expression in CHO

A master cell line from the glycosylation mutant CHO Lec3.2.8.1 cell line containing an RMCE cassette was previously developed in our group. The cultivation, integration of genes via recombinase-mediated cassette exchange and isolation of single production cell clones were performed as described (12). Small scale protein production with the expression cell line was performed by cultivation in 300 mL suspension cultures for 5 days. The production of ECD-mTLR2 in CHO Lec3.2.8.1 was performed by continuous cultivation in a membrane-aerated 2.5-L bioreactor in perfusion mode using a total volume of 40 L culture medium [22]. The supernatant was concentrated by ultra- and diafiltration (Millipore ProFlux M12 with Pellicon TFF system) prior to affinity chromatography.

### Transient protein production in Baculovirus-Infected Insect Cells

For protein expression, recombinant bacmids were generated using the Tn7 transposition method in bacmids of the MultiBac (MB) [23] or EMBacY (MBY) system [18], respectively and both pFlpBtM-I and pFlpBtM-II as donor vectors. MBY bacmids include a YFP-gene as a marker for monitoring infection kinetics. Sf21 (DSMZ #ACC 119) and BTI-Tn-5B1-4 (High Five, Invitrogen) suspension cultures were cultivated in ExCell420 (SAFC) on orbital shakers at 100 r.p.m. at 27°C using a 2.5 cm orbit. For transfection 0.75×10^6^ cells/well were seeded into 6-well-plates. For each transfection 10µl Superfect (Qiagen #301305) and 5µl isolated bacmid were diluted in 100 µl serum-free medium and incubated for 20 min at RT. The culture medium covering the adherent cells was replaced by the transfection mixture. After 2 h the transfection mixture was aspirated and 2 ml medium were added. Virus supernatant was harvested 3–5 days post transfection depending on the development of the YFP response. After virus amplification the titers were determined by plaque assays. For protein expression suspension cultures with an initial cell density of 0.5×10^6^ cells/mL were infected using MOIs between 1–3 or 10 vol% of V1 Virus Stock. Infection kinetics were monitored by the determination of the growth curves, cell diameter and percentage of fluorescent cells.

### Recombinant Protein Purification

Intracellular model proteins were isolated from cell pellets after cell lysis in 50 mM Na-Phosphate, 300 mM NaCl, 5 mM Imidazol, 0,5% NP40, 3 mM β-mercaptoethanol supplemented with 10 µg DNaseI, Roche complete mini protease inhibitor tablet without EDTA. Supernatants and cell lysates were filtrated using Minisart 0.45 µm syringe filters (Sartorius). Purification of the model proteins was performed using the Profinia System (BioRad) via Ni-NTA IMAC for the purification of fluorescent model proteins and mTLR2. Protein A Affinity Chromatography was used for isolation of scFv-hIGg-protein constructs. Analysis of protein expression and purification was performed by SDS-PAGE and Western blots.

### SDS-PAGE and Western Blotting

All samples containing recombinant proteins were analyzed by 12% SDS-PAGE. For the specific detection of mCherry and ECD-mTLR2 western blots were performed using anti-Histag mouse monoclonal antibody (Novagen #70796, dilution 1∶1000) and AP-conjugated Anti-Mouse IgG (H+L) (Promega #S372B). Goat-anti-human IgG (H+L)- AP conjugate (Promega #S3821) was used for detection of scFv-Fc constructs.

## Results

### Generation of pFlp-Bac-to-Mam

**Figure 3:**
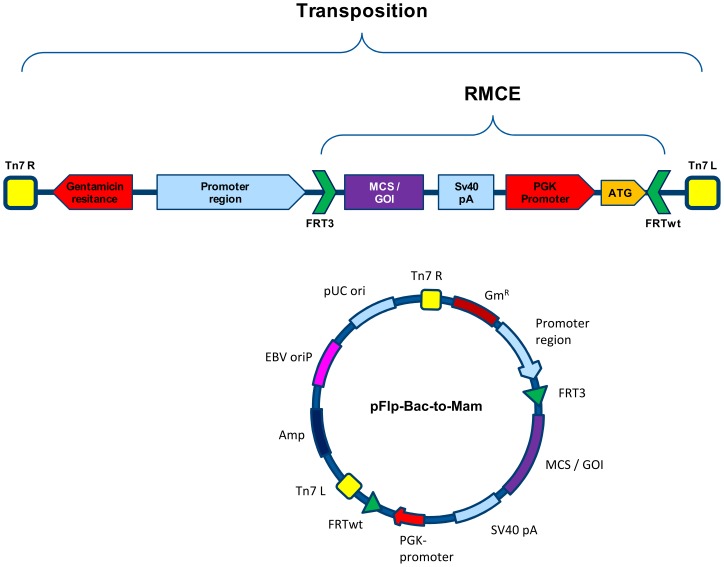
pFlpBtM architecture. The multi-purpose vector pFlpBtM contains elements necessary for the use as a donor vector for Flp-recombinase mediated cassette exchange (FRT  =  Flp recombinase target sites) and Tn7-transposition sequences for the generation of recombinant bacmids. FRT-sites flank the region which is integrated into the RMCE locus in the master cell line. It contains the MCS and a downstream PGK-promoter for a selection trap method to screen for recombined cell clones. A larger section of the plasmid is integrated into the bacmids by TN7-based transposition. It contains the promoter region and a gentamicin-resistance gene for the selection of recombinant bacmids. The backbone of pFlpBtM-II additionally contains an Epstein-Barr virus oriP for increased nuclear transport and episomal replication in EBNA positive cell lines.

The construction of the novel multi-purpose donor- and expression vector pFlp-Bac-to-Mam (pFlpBtM) is described in detail in the Materials and Methods section. The schematic architecture of the pFlpBtM backbone is shown in Figure 3. It comprises genetic elements necessary for the generation of stable mammalian producer cell lines by RMCE and for Tn7-based transposition baculovirus expression vectors in a single plasmid. Additionally, the integration of the Epstein-Barr-Virus oriP in the backbone of pFlpBtM-II renders the vector also very suitable for transient expression in optimized cell lines like HEK293-6E [2]. To demonstrate its applicability for the different expression methods, the genes of the three model proteins were subsequently integrated and their expression was evaluated.

### Expression of intracellular model protein mCherry

**Figure 4:**
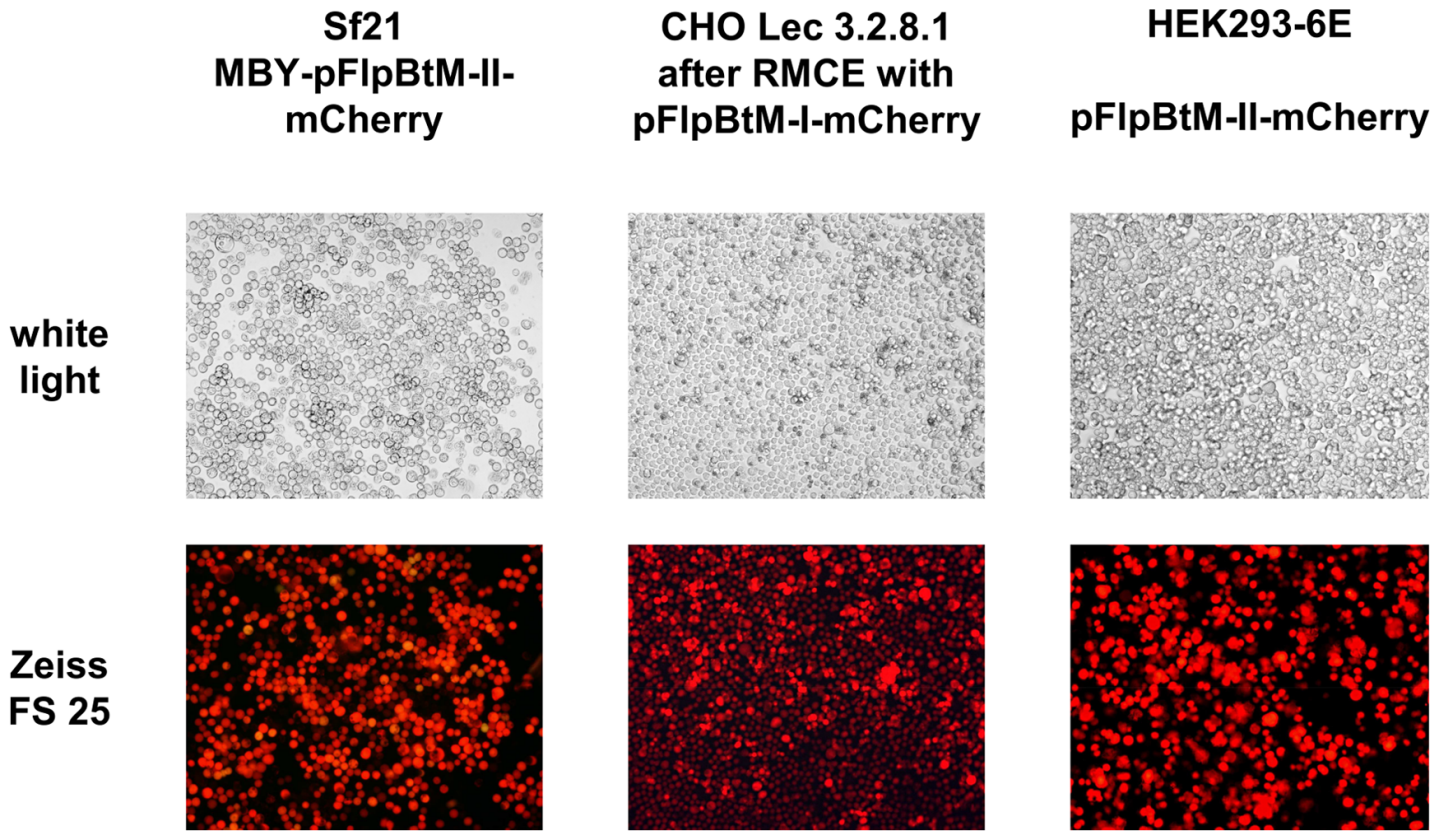
Fluorescence microscopy pictures of cells producing mCherry. Infection rates of more than 95% were achieved in Sf21 with pFlpBtM-derived virus producing mCherry (left). Likewise, transfection rates of more than 80% percent were confirmed by flow cytometry in transient transfection of HEK293-6E with pFlpBtM-II-mCherry (right). Homogenous expression of the model protein in stable isolates of CHO Lec3.2.8.1 mCherry-producer cell lines were monitored for more than 3 months upon cassette exchange using pFlpBtM as donor vector (middle).

The vector pFlpBtM-II-mCherry-His_6_ was used for transient expression in HEK293-6E and for the generation of recombinant Baculovirus. Establishing stable CHO Lec3.2.8.1 producer cell lines by RMCE was performed using pFlpBtM-I-mCherry-His_6_. The successful expression of mCherry in each system was monitored by flow cytometry and fluorescence microscopy. Average transfection rates of >70% were achieved by transient expression in HEK293-6E cells. Likewise, more than 90% of the Sf21 cells were identified as mCherry-positive 72 h post infection with recombinant baculovirus. Upon cassette exchange with pFlpBtM-I-mCherry-His_6_ as donor vector, several neomycin resistant CHO cell clones stably expressing mCherry were isolated and propagated for more than 16 weeks with stable expression without antibiotics selection (Figure 4).

Cell pellets of 50 mL cultures of each expression system were harvested by centrifugation and lysed with detergents and sonication. Recombinant mCherry-His_6_ protein was captured by Ni-IMAC. Average yields of the model proteins are summarized in Table 1. The results show that both plasmid based transient expression in HEK293-6E and baculoviral expression in insect cells are equally eligibly for the production of the intracellular model protein mCherry. Due to the slightly higher yields and the significantly lower practical effort, the plasmid based transient expression in HEK293-6E is advantageous compared to the more laborious BEVS in this case. However, if repeated experiments or large-scale expression (>1 L) is desired, the BEVS might be superior, since the recombinant baculovirus can be efficiently stored for long time preservation without loss of infectivity as cryo stocks of baculovirus infected insect cells (BIIC) [24]. Firstly, this technique allows for unlimited replications of the experiments with marginal batch-to-batch variability and effective scale up. Secondly, the baculoviral expression is cheaper due to the high costs of the mammalian cell culture medium and preparation of large batches of endotoxin free DNA necessary for large-scale transient transfection. In contrast to both described transient expression methods, only about 8 mg/L mCherry were produced by stable genomic expression of mCherry upon Flp mediated exchange into the CHO Lec3.2.8.1 RMCE master cell line. This low expression level is most likely caused by differences in gene dose. The transient expression systems allow multi-copy transfection, whereas the expression of the mCherry-His_6_ transgene in the generated stable CHO Lec3.2.8.1 producer cell line is dependent on the single gene copy nature of the Flp mediated cassette exchange reaction.

**Table 1:** Average product yields in different expression systems using pFlpBtM as a donor or expression vector.

Volumetric yields [mg/L]			
Protein	HEK293-6E	BEVS	CHO Lec3.2.8.1
mCherry	52±6	42±7	8
ECD mTLR2	n/d	n/d	0.8±0.1
scFc-hIgG1Fc	90±30	1.7±0.9	-

### Expression of scFv-Fc as secretory model protein

**Figure 5:**
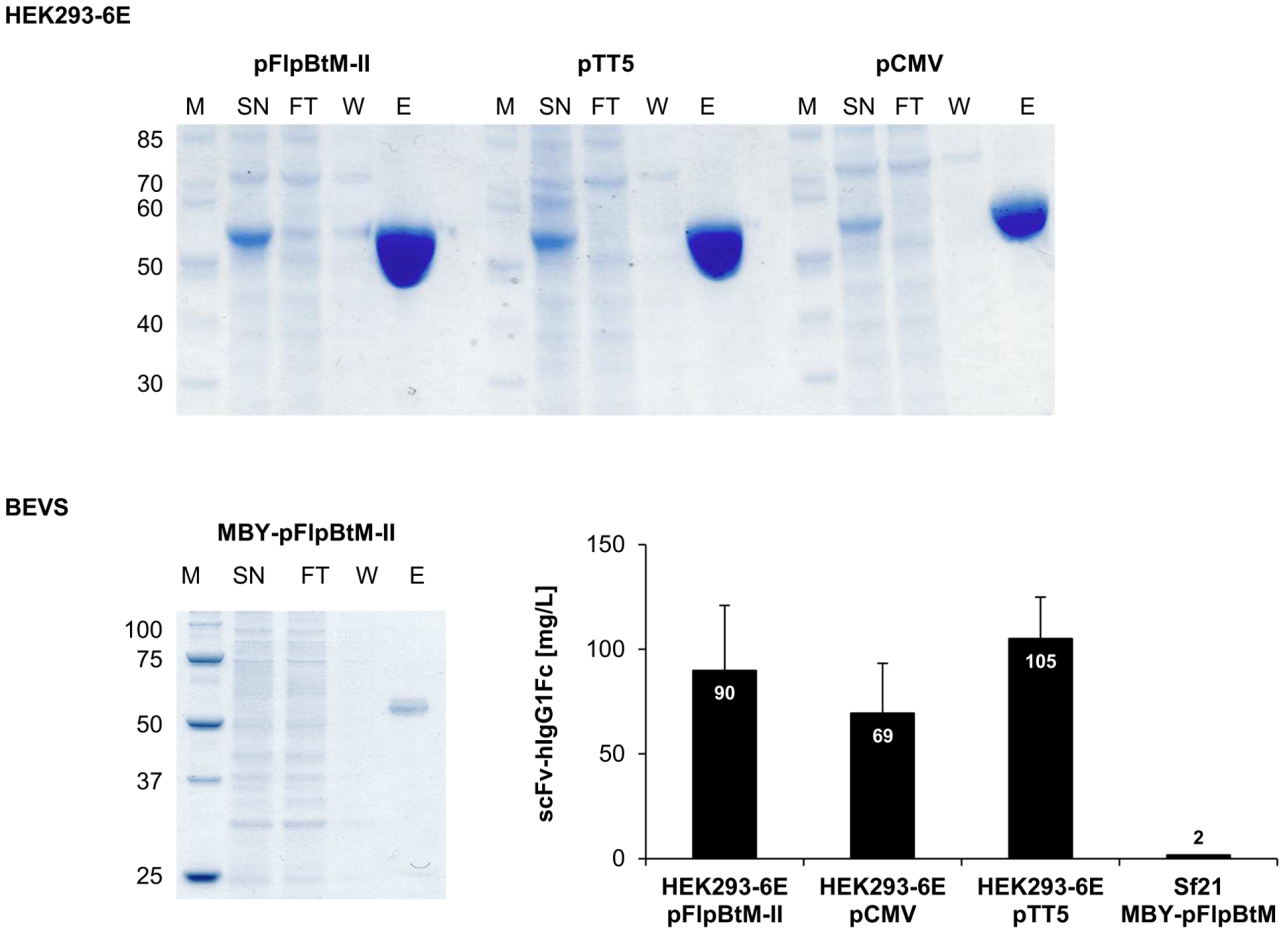
Production of scFv-Fc in HEK293-6E and Sf21. SDS-PAGE of protein-A affinity chromatography purified fractions from supernatants of transient expression of scFv-Fc in HEK293-6E and in BEVS (SN  =  supernatant, FT  =  flowthrough, W  =  wash, E  =  eluate). Comparison of average yields of purified scFv-Fc expressed in HEK293-6E using different expression vectors and expressed in BEVS.

Expression of scFv-Fc using pFlpBtM-II was performed in BEVS and HEK293-6E. The construct pFlpBtM-II-scFv-Fc was generated by ligation of the scFv-Fc Fragment from the plasmid pCMV-scFv-Fc into linearized pFlpBtM-II. Since scFv-Fc represents a model protein for therapeutic glycoproteins the expression in the CHO Lec3.2.8.1 glycosylation mutant cell line with its simple mannose-type *N*-glycosylation was omitted. Alternatively, we compared the expression characteristics of pFlpBtM-II in HEK293-6E with alternative expression vectors available for this system. Hence, the scFv-Fc construct was integrated into pTT5, one of the best expression vectors for the HEK293-6E cells. Parallel scFv-Fc expression tests were also performed comparing pFlpBtM-II and pTT5 with the parental pCMV-scFv-Fc vector. The results are summarised in Figure 5 and Table 2.

**Table 2:** Average volumetric yields of scFv-Fc construct in HEK293-6E using different expression vectors.

Volumetric yields [mg/L]			
	pFlpBtM-II	pCMV	pTT5
scFv-hIgG1Fc	90±30	70±24	105±20

The parental pCMV-scFv-Fc vector (4912 bp) without an EBV oriP led to an average production of 69 mg/L scFv-Fc. Using pFlpBtM-II and pTT5 derived constructs, both carrying the EBV oriP, yields increased to more than 100 mg/L. The average scFv-Fc-yields were 90 mg/L for pFlpBtM-II and 105 mg/L for pTT5, respectively. The 40% increase in expression is most likely due the presence of the oriP as replication origin. Gene dose effects most probably cause the small but reproducible difference of 14% between the oriP containing vectors. Since pFlpBtM-II harbours genetic elements for its use as a multi-host expression vector the plasmid has a size of 6865 bp and is thus about 40% larger than pTT5 (4401 bp). Correspondingly the number of gene copies per µg transfected DNA is lower. The reduction in gene dose for pFlpBtM-II is more severe then the difference in transient expression level. These results show the full applicability of pFlpBtM-II in the transient HEK293-6E (EBNA) expression system.

To evaluate the expression of scFv-Fc in BEVS recombinant MBY-bacmids were generated using pFlpBtM-scFv-Fc as donor vector. Following virus production and titer-determination several expression tests were performed in Sf21 and High Five cells with an MOI of 2 or 10% untitered V1 virus, respectively. Expression and secretion of the protein was identified by SDS-PAGE analyses. However, subsequent purification via Protein A affinity chromatography revealed yields of less than 4 mg/L. These results indicate that the baculovirus expression vector system is not the optimal system for high yield production of scFv proteins.

### Expression of the ECD of murine TLR2

**Figure 6:**
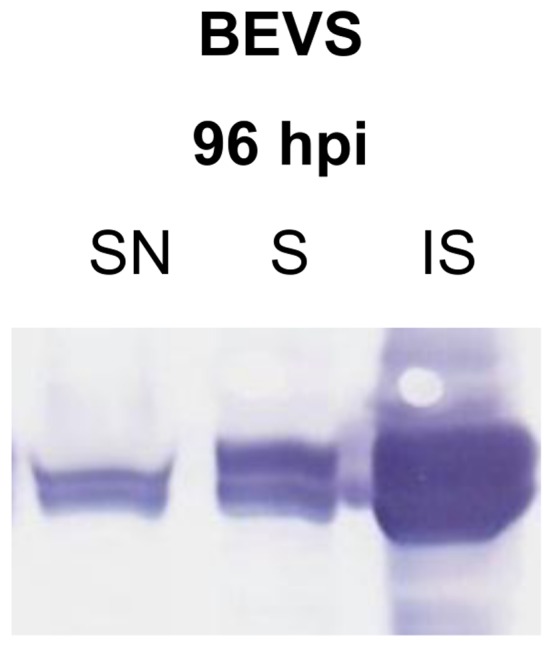
Expression profile of ECD mTLR2 in BEVS. Westen Blot analysis of the culture supernatant and intracellular fractions of Sf21 infected with recombinant pFlpBtM-II derived baculovirus producing ECD-mTLR2. Significant amounts of recombinant protein accumulate intracellularly as insoluble material due to compromised folding and secretion capability of virus infected cells. (SN  =  supernatant, S  =  intracellular soluble fraction, IS  =  intracellular insoluble fraction).

In previous work, expression of the ECD of mTLR2 has been performed in insect cells [25], [26]. However, no exact expression characteristics or yields of soluble protein were presented in these reports. In our lab yields from 0.3 to 1 mg/L were achieved upon purification of recombinant mTLR2 from insect cell culture supernatants using an mTLR2 expression construct comprising the first 593 amino acids of the ECD cloned into a pFastbac donor plasmid (Invitrogen) (data not shown). The expression of ECD mTLR2 in the BEVS was reproduced after generating recombinant viruses using pFlpBtM-II-mTLR2 as donor vector for Tn7 based transposition. As observed before, significant amounts of the protein remained in the cytoplasm and even accumulated in the insoluble fraction (Figure 6).

To enhance both yield and quality of the recombinant protein, alternative expression strategies were evaluated using pFlpBtM. Analysis of transient expression of ECD-mTLR2 was performed in HEK293-6E cells. However, as opposed to the results of mCherry and scFv-Fc, expression and secretion of ECD-mTLR2 in HEK293-6E cells was not improved compared to the BEVS. In fact, both solubility of the heterologous protein and yield of purified protein from the culture supernatant was inferior to the observations in the BEVS. Western blot analysis revealed, that the ECD-mTLR2 protein accumulated almost solely in the insoluble fraction and only minor amounts of secreted soluble ECD-mTLR2 could be enriched by IMAC from culture supernatants. The resulting protein concentration in the elution fraction was too low for an accurate determination of the yield (data not shown).

**Figure 7:**
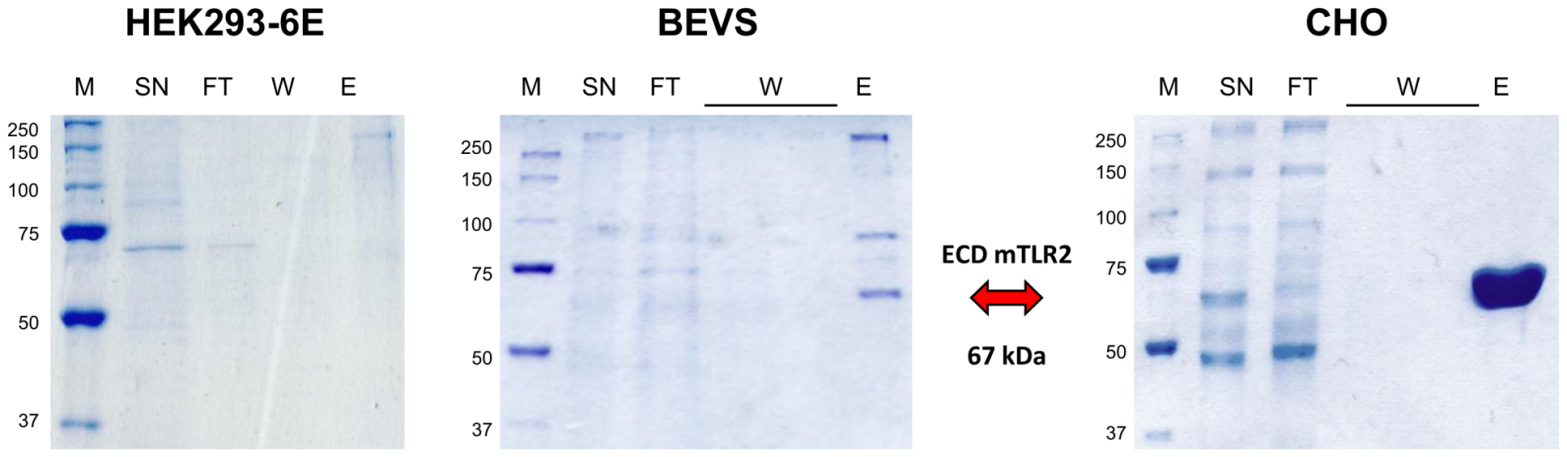
ECD mTLR2 production. SDS-PAGE of IMAC fractions of the secreted model protein from culture supernatant upon expression in HEK293-6E transfected with pFlpBtM-II-ECDmTRL2, in the BEVS using an MBY-pFlpBtM-II-ECDmTRL2 virus and in stable CHO Lec3.2.8.1 producer cell lines generated via RMCE with pFlpBtM-II-ECDmTLR2. Purification of the target protein from concentrated supernatant of CHO fermentation yielded substantial amounts of homogenous material. In contrast, significant traces of host cell protein are co-purified from insect cell supernatants due to virus-mediated cell lysis. Protein yields captured from HEK293-6E supernatant are too low to be visible on SDS-PAGE gels. SN  =  supernatant, FT  =  flow through, W  =  wash, E  =  eluate, M  =  Precision Plus Protein Standard [kDa] (Biorad).

The RMCE method with pFlpBtM-II-ECD-mTLR2 was used to isolate a clonal CHO producer cell line stably expressing ECD-mTLR2. As the yield in expression was expected to be low using single copy expression RMCE cell lines, a stable producer cell line was cultivated in continuous perfusion culture in a bioreactor using an overall perfusion volume of 35 L. The ECD-mTLR2 protein was purified from concentrated culture supernatant after ultra-and diafiltration using IMAC. Three independent experiments resulted in an average yield of 0.8 mg purified Protein/L. These yields are comparable with those achieved from the expression of ECD-mTLR2 in the BEVS. However, capturing ECD-mTLR2 from cell culture supernatants of the CHO producer cell lines provided a higher purity with less contamination compared to the expression in BEVS, where a significant contamination by host cell proteins is observed due to cell lysis (Figure 7).

## Discussion

**Figure 8:**
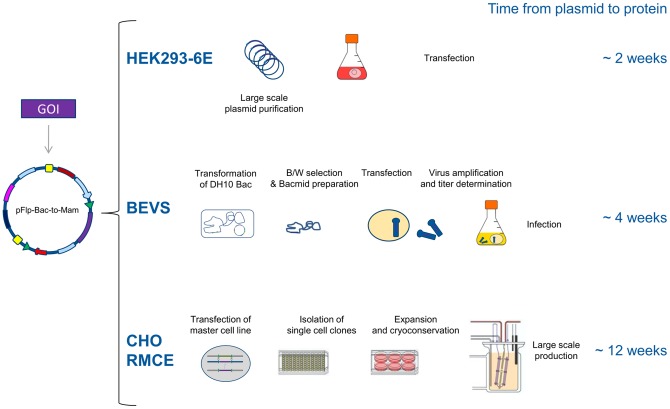
Overview of the applicability of the pFlpBtM vector for different expression strategies. Upon integration of the target gene into pFlpBtM the vector can be used for transient expression in HEK293-6E, as a donor vector for Tn7-transposition based generation of recombinant bacmids for the BEVS and to generate stable producer CHO cells lines by RMCE.

The initial screen for protein variants to identify expressible constructs and to determine the optimal expression host for a given protein is the most time consuming process in a protein production pipeline using eukaryotic expression systems. To address this, we have successfully established a single expression vector for several eukaryotic hosts that allows direct analysis in transient gene expression (TGE), baculoviral expression (BEVS) and stable genomic expression methods (RMCE) in mammalian and insect cell lines. The versatile pFlp-Bac-to-Mam expression vectors allow a multiparallel approach comprising fast screening of expressible constructs without the need for recloning in the above-mentioned expression systems. We implemented for the first time a combination between the potent RMCE system for fast generation of stable producer cell lines in 8 weeks, the well-known Tn7-transposase based generation of recombinant bacmids for baculoviral expression in insect cells and transient transfection in EBNA1-expressing mammalian HEK293-6E cells. Since pFlpBtM can be used for both, fast transient and stable genomic expression in mammalian cells as well as a donor vector for the generation of recombinant bacmids it accelerates the initial screening for expressible constructs and the most suitable host for any given protein (Figure 8).

In a comparative test expression with model proteins of three different protein classes, including a secretory scFv-Fc, the ECD of murine Toll like receptor 2 and the intracellular protein mCherry, the different expression strategies and hosts were evaluated. Each protein showed different expression characteristics in the tested hosts. Thereby it was possible to determine the optimal expressions strategy for each model protein. The intracellular yield of mCherry varied within one log scale between 8 mg/L in stable expression in the RMCE-CHO cell line and 52 mg/L in transient expression both in the BEVS and HEK293-6E system. Thus, the stable expression in RMCE based cell lines has to be considered as less favourable for the intracellular expression of the mCherry protein compared to expression with higher copy number in viral and plasmid-based transient systems.

Parallel TGE of the scFv-Fc model protein in HEK293-6E was performed to benchmark the expression capability of pFlpBtM-II compared to the conventional pCMV vector and pTT5 which has been the dedicated expression plasmid for this cell line. Due to its multiple genetic elements pFlpBtM-II-scFv-Fc is 40% larger than pTT5-scFv-Fc and 30% larger compared to pCMV-scFv-Fc. Despite the resulting noteworthy decrease of the gene dose, TGE in HEK293-6E using pFlpBtM-II yielded an average amount of 90 mg/L of purified scFv-Fc, which corresponds to 85% of the yield achieved with pTT5. Moreover, the concentration of captured antibody was 30% higher compared to pCMV, which is lacking the EBV-oriP. This indicates that the size of the vector is less important than the presence of the oriP replication origin. These findings demonstrate the suitability of pFlpBtM-II as alternative expression plasmid for transient expression in HEK293-6E. The slightly lower expression capability compared to the smaller, optimised expression plasmid pTT5 is negligible compared to the advantage in high-throughput construct optimisation and expression screening and its versatile option for direct transfer to the production scale in different expression systems. Upon viral expression in Sf21 or Hi5 an average of 2 mg/L of the scFv-Fc could be captured from insect cell supernatants by protein A chromatography. This significantly lower yield compared to TGE in HEK293-6E corresponds to the titers previously reported upon expression of in the BEVS [27], [28]. The results show that plasmid based transient expression in HEK293-6E is superior to the baculovirus expression vector system for antibody constructs.

For ECD-mTLR2, evaluation of the different expression methods enabled a significant improvement of both solubility and purity of the target protein by shifting to large-scale stable expression instead of baculoviral expression. Obviously, the lower rate in stable genomic expression is compensated by the quality of the system in proper folding and secretion of the ECD-mTLR2 protein. As a stable producer cell line has been generated the yields and the significantly higher quality can be reproducibly achieved. Thus, stable genomic expression of difficult to express proteins as the extracellular domain of mTLR2 is superior to transient or viral expression strategies.

The results shown in this paper demonstrate that a profound screening for the optimal expression system for any given target protein is crucial both for the success and efficiency of a recombinant protein expression. Subsequent to screening for the best expressible constructs, pFlpBtM enables for directly shifting to large-scale production with the proper construct in the optimal system avoiding time-consuming recloning steps. Using the presented integrated expression platform each of the model proteins could be expressed in sufficient amounts for subsequent structural and biophysical analyses even without further process development. The fast screening ability offered by the pFlpBtM vector is of major advantage in protein expression for structural biology. In this field, high flexibility to meet the needs of fast changing projects and diverse target proteins is far more important than industrial scale yields achieved by time consuming process development strategies. Hence, the versatile multi-host expression vector is the new standard eukaryotic expression vector at the Helmholtz Protein Sample Production Facility. By the ease of modification of the multiple cloning site, the vector can be further adapted to individual cloning or purification strategies (e.g. by the integration of LIC sequences, restriction sites or affinity tags), as well as expanded to other expression systems like *Pichia pastoris*. The pFlpBtM vector as part of our multi host expression system (mHost-XS) is a valuable tools to widen the bottleneck in production of mammalian proteins for biochemical and structural analyses.
